# The Impact of Negative Emotion on Visual Working Memory Under Excessive Cognitive Load: The Role of the Resource-Allocation Stage and Location Information

**DOI:** 10.3390/bs16101849

**Published:** 2026-10-09

**Authors:** Di Zhang, Taiwei Tan, Tengyi He, Fangfang Long

**Affiliations:** 1 Key Laboratory of Brain Function and Brain Disease Prevention and Treatment of Guizhou Province, Guiyang 550025, China; 2 School of Psychology, Guizhou Normal University, Guiyang 550025, China; 3 School of Psychology, Capital Normal University, Beijing 100048, China; 2263501018@cnu.edu.cn

**Keywords:** visual working memory, negative emotion, working memory capacity, excessive cognitive load, location information

## Abstract

Visual working memory (VWM) enables people to temporarily store important information for use in subsequent tasks. Previous studies have indicated that a negative emotional state can influence the processing of VWM; however, the direction of this influence and its underlying cognitive mechanisms remain contentious. The present study investigated the impact of negative emotion on VWM, with a particular focus on how this association was manifested across cognitive-load conditions, theoretically defined resource-allocation stages, and location-information conditions. Across two experiments, negative emotion was found to exert a bidirectional effect: it impaired VWM performance under low cognitive load, whereas it produced a facilitative effect under excessive cognitive load. Importantly, this facilitative effect emerged only during the early stage of resource allocation and when the locations of memory items and probe items were congruent. Under these specific conditions, negative emotion increased VWM capacity (K = 3.86, SE = 0.23) compared with the neutral condition (K = 3.32, SE = 0.22), whereas this facilitative effect disappeared when location congruence was disrupted. These findings demonstrate that the effects of negative emotion on VWM depend on the interaction between emotional states, cognitive load, and location-information availability. This provides important evidence for understanding the boundary conditions under which emotion modulates cognitive function, and offers theoretical guidance for load-sensitive and emotion-aware cognitive system design in complex information-processing environments.

## 1. Introduction

Visual working memory (VWM) screens and filters the stimuli entering the visual field, retains information temporarily, and makes it available for subsequent cognitive processing after the visual input has disappeared (Cowan, 2011; Luck & Vogel, 1997; Vogel et al., 2005). VWM capacity is typically limited to about 3–4 items, and this capacity level is closely related to the performance of various cognitive activities (Cowan, 2001; Unsworth et al., 2015). Although an individual’s VWM capacity is relatively stable, it can still be influenced by factors such as individual traits, environment, and emotional state (Luck & Vogel, 2013; Zhang et al., 2017). As a form of psychological activity mediated by the individual’s needs, emotion always accompanies and influences the individual’s cognitive processing (Droit-Volet & Meck, 2007; Kissler et al., 2007; Koster et al., 2007). In cognitive task contexts, negative emotion can be regarded as interfering information (Yang et al., 2021). According to the Processing Efficiency Theory (PET), negative emotion occupies working-memory resources, thereby reducing capacity and impairing task performance—that is, individuals require more resources to cope with the emotion rather than focusing on the task (Eysenck & Calvo, 1992; Li et al., 2024).

Some findings indicate that negative emotion impairs VWM performance. Figueira et al. (2017), in an experiment combining emotional picture induction with a change detection task, found that under a negative emotional state, the number of task-relevant items participants could maintain was significantly reduced and task performance decreased. Long et al. (2020) further corroborated this finding in an experiment that combined emotional picture induction with a recall report task, demonstrating that negative emotion significantly reduced the number of memory items participants were able to maintain. Further studies have shown that anxiety, stress, and other negative emotions all significantly impair individuals’ VWM performance (Hunter & Shields, 2024; Ward et al., 2020; Ye et al., 2024). In addition, meta-analyses and review studies consistently indicate that task-irrelevant negative emotional distractors significantly impair VWM performance and capacity (J. Chen, 2025; Schweizer et al., 2019). Colliot et al. (2025) also found that negative (versus neutral) images reduced memory performance in a complex span task even when participants were not required to process the emotional images, indicating that the mere presence of negative stimuli involuntarily captures attention and consumes resources needed for working-memory maintenance. Extending this to the encoding stage, Wu et al. (2026) reported that a negative emotional state left target processing intact but weakened the active suppression of irrelevant information during encoding (as indexed by a smaller distractor positivity), such that distractors intruded into and occupied limited VWM capacity, indicating that negative emotion degrades VWM primarily by disrupting inhibitory control over distractors rather than by hindering target selection.

However, other studies have found that negative emotion has no effect on VWM capacity (Ribeiro et al., 2023; Souza et al., 2021; Valenti et al., 2021; Xie & Zhang, 2016). For example, by using a recall report task in which they manipulated negative, neutral, and positive emotional conditions and asked participants to memorize color or shape stimuli, a previous study found no significant difference in VWM capacity across the three emotional conditions (Xie & Zhang, 2016). Subsequent research found that negative emotion facilitates the VWM capacity of individuals with high VWM capacity while impairing that of individuals with low VWM capacity (Zhang et al., 2017). These lines of evidence suggest that the effect of negative emotion on VWM capacity is not stable, and its underlying mechanism remains unclear.

Cognitive load refers to the extent to which cognitive resources are occupied during task processing, and is commonly manipulated by adjusting the number and order of items in a memory sequence (Ahmed & de Fockert, 2012; F. G. W. C. Paas & Van Merriënboer, 1994; Sweller, 1988). The Cognitive Load Theory (CLT) holds that individuals’ cognitive performance is strictly constrained by working-memory capacity (Skulmowski & Xu, 2021; Sweller, 2023; Sweller et al., 1998); when task complexity is high, or information presentation is unreasonable, excessive cognitive load rapidly depletes working-memory resources and weakens information-processing efficiency and maintenance ability (F. Paas & van Merriënboer, 2020). Studies have shown that cognitive load significantly affects the allocation of cognitive resources in VWM processing (Balestrieri et al., 2021; Bays et al., 2009; Fockert et al., 2001; Kelley & Lavie, 2010); increased cognitive load not only crowds out the resources for maintaining visual representations but also interferes with the inhibition of irrelevant information, leading to a reduction in effective capacity (Cowan, 2001; Vogel et al., 2005; Ye et al., 2024). Thus, cognitive load indirectly determines VWM capacity and performance by regulating the efficiency of working-memory resource allocation—that is, the higher the task load, the fewer effective resources the individual can devote to maintaining and manipulating visual information.

The aforementioned studies indicate that both negative emotion and cognitive load can affect VWM performance and capacity by influencing the allocation of VWM resources. During cognitive processing, these two factors often co-occur and exhibit an interaction. Existing research has shown that when cognitive resources are limited and cognitive load rises markedly, individuals tend to prioritize allocating more resources to the processing of task-relevant information while reducing resource investment in distractor processing, thereby limiting the processing of irrelevant stimuli and indirectly reducing their interference with the target task (Gunduz & Ozkan Ceylan, 2024; Lavie et al., 2014; P. Liu et al., 2018). Moreover, the Mere Resource Hypothesis (MRH) holds that emotion processing and cognitive tasks share limited resources (Kron et al., 2010); when negative emotion serves as a distractor (Yang et al., 2021), high cognitive load can reduce the intensity of negative-emotion processing (DeFraine, 2016; Mikels & Reuter-Lorenz, 2019). Rozovsky et al. (2025) provided converging neural evidence for this account. Using dynamic causal modeling in an emotional N-back task, they found that when high working-memory load was combined with simultaneous emotion regulation, integration between the default mode and central executive networks was reduced to preserve task performance—suggesting that elevated cognitive load suppresses emotional processing within large-scale brain networks. Convergent behavioral evidence is provided by Sun et al. (2026), whose EEG recordings during a Sternberg working-memory task showed that threat-related stimuli modulated theta power under low load in the high-anxiety group, but this effect vanished under high cognitive load. These findings suggest that increased load suppresses the neural processing of emotional information, thereby shielding visual working memory from emotional interference.

These theories jointly reveal that negative emotion and cognitive load may interactively influence VWM capacity through a competitive resource mechanism. Existing studies have shown that under low cognitive load, negative emotion impairs VWM capacity, whereas under high cognitive load, negative emotion has no effect on VWM performance and capacity. For example, Chouinard-Gaouette and Blanchette (2025) induced emotion by having participants watch negative or neutral videos before completing an N-back task. The results showed that under low cognitive load (2-back), the P300 amplitude of the negative-video group was significantly lower than that of the neutral-video group, a phenomenon indicating that negative emotion interfered with the neural processing of VWM; under high cognitive load (3-back), there was no significant difference in P300 amplitude between the two groups. This result, revealed at the neural level, is consistent with behavioral findings. Some behavioral studies have shown that under low cognitive load, negative emotion impairs VWM performance, whereas under high cognitive load, negative emotion has no effect on VWM performance (Figueira et al., 2017; Long et al., 2020; Valenti et al., 2021; Xie & Zhang, 2016; Ye et al., 2024). This dynamic modulation can be better understood in light of the Perceptual Load Theory (PLT): at low load, residual resources process distracting information, whereas at high load, attentional resources are exhausted by the target task, thereby blocking negative-emotion interference (Lavie, 2005; Scalf et al., 2013). It can thus be seen that negative emotion and cognitive load interact in influencing VWM capacity, and the direction and strength of this interaction depend on the extent to which the task occupies resources.

Existing research has also found that the interaction between negative emotion and cognitive load on VWM performance is related to the resource-allocation stage during the consolidation phase (Guo et al., 2023; Q. Liu et al., 2024; Ren et al., 2025; Xie et al., 2022). For example, Long et al. (2020) found that under high cognitive-load conditions, negative emotion had no effect on VWM performance when stimuli were presented for 200 ms, corresponding to the early stage of consolidation, whereas negative emotion impaired VWM performance when stimuli were presented for 500 ms, corresponding to the late stage of consolidation. This temporal distinction was derived from the two-phase model of VWM resource allocation proposed by Ye et al. (2017). The model proposes that resource allocation during the early consolidation phase is predominantly stimulus-driven and involuntary, whereas, after sufficient consolidation time, allocation may enter a later phase in which it can be more flexibly adjusted according to task demands. In Ye et al. (2017), 200 ms and 500 ms presentation durations were used to operationalize conditions preferentially associated with the early and late phases, respectively. Long et al. (2020) subsequently adopted this framework to examine whether negative emotional states differentially influenced VWM under short and long presentation durations. Accordingly, the present study used 200 ms and 500 ms memory-array presentation durations as operational indicators of conditions theoretically associated with the early and late resource-allocation stages.

In summary, existing research has jointly revealed, at both behavioral and neural levels, the interaction between negative emotion and cognitive load in VWM performance and capacity and its temporal characteristics; however, relevant conclusions mainly focus within the range of ordinary cognitive load, and there remains a lack of systematic investigation into the influence under conditions exceeding the individual’s VWM capacity ceiling (i.e., excessive cognitive load). In real cognitive activities—such as high-pressure decision-making, medical operations, and emergency response—individuals often need to maintain task performance while experiencing both negative emotion and information overload (Souchet et al., 2022). According to the PLT, when task demands approach or exceed the system’s capacity ceiling, limited resources are fully devoted to core processing, thereby inhibiting the processing of emotional information. It is thus inferred that as cognitive load increases, the impairing effect of negative emotion on VWM capacity gradually weakens and disappears under excessive cognitive load. Based on this, the present study employed an emotion-induced change detection task, with three cognitive-load conditions—low cognitive load (two items), high cognitive load (four items), and excessive (six items)—as well as early resource-allocation (200 ms) and late resource-allocation (500 ms) conditions, to investigate the interaction between negative emotion and cognitive load on VWM performance and its temporal characteristics.

## 2. Experiment 1

### 2.1. Purpose

To verify the effect of negative emotion on VWM and the joint effect of negative emotion and cognitive load on VWM at different stages of resource allocation, Experiment 1 was conducted. The hypotheses of Experiment 1 were as follows: (1) VWM performance under the negative emotional condition is significantly lower than under the neutral emotional condition; (2) as cognitive load increases, VWM performance under the negative emotional condition decreases significantly; (3) under excessive cognitive load, the impairing effect of negative emotion on VWM performance is weakened or disappears; and (4) the effect of negative emotion on VWM performance differs significantly between the early and late stages of resource allocation.

### 2.2. Method

#### 2.2.1. Participants

Forty adult students from Guizhou Normal University were randomly recruited. An a priori sample-size calculation was conducted using G*Power 3.1 for a repeated-measures ANOVA. The calculation assumed a medium effect size of Cohen’s f = 0.25, an alpha level of 0.05, and a target power of 0.85, yielding a minimum required sample size of 21 participants. The sample included 21 females, with a mean age of 22.15 ± 1.72 years. Data from five participants whose accuracy was below 60% were excluded (Long et al., 2020), leaving data from 35 participants for the final analysis. All participants had normal or corrected-to-normal vision, no color blindness or color weakness, and were right-handed. They received monetary compensation upon completion of the experiment. This study was approved by the Committee for the Protection of Human Subjects at the School of Psychology, Guizhou Normal University (approval number: GZNUPSY.N.202312E [0021]).

#### 2.2.2. Stimuli

All participants completed the experiment in a quiet, isolated room at a viewing distance of 60 cm from the screen. This study adopted a change detection task involving emotional arousal (Figueira et al., 2017; Long et al., 2020). The experiment was programmed in E-Prime 2.0, and all stimuli were presented on a 14-inch monitor (1024 × 768 pixels). For the emotional pictures, 40 negative and 40 neutral images were selected from the revised International Affective Picture System (IAPS) and converted to grayscale (Lang et al., 1997; Long et al., 2020). Although these images were originally colored, grayscale conversion was used to reduce the potential interference of preceding pictures with the subsequent VWM task (Xie & Zhang, 2016). Before the formal experiment, 50 participants were randomly recruited to rate the valence and arousal of the selected pictures. The change detection task used colored squares (1°) as memory stimuli. All stimuli were presented within a rectangular region (11°) against a gray background (RGB: 120, 120, 120). The position of the stimuli was randomized within the rectangular region on each trial. Seven colors were used: red (RGB: 240, 0, 0), green (RGB: 0, 130, 0), orange (RGB: 200, 200, 0), blue (RGB: 0, 0, 200), cyan (RGB: 31, 165, 157), black (RGB: 0, 0, 0), and purple (RGB: 120, 0, 120).

#### 2.2.3. Design and Procedure

The experiment employed a 2 (resource allocation stage: early (200 ms), late (500 ms)) × 2 (emotional state: negative, neutral) × 3 (cognitive load: low (2 items), high (4 items), excessive (6 items)) within-subjects design. For the change detection task, response times, accuracy, and memory capacity K were computed, where

$$K=\frac{S\times (H-\mathrm{FA})}{\left(1-\mathrm{FA}\right)},$$

with S denoting the number of memory items presented, H the hit rate, and FA the false alarm rate (Pashler, 1988). The experiment comprised 240 trials, divided into two blocks of 120 trials each. Before the formal experiment, each participant completed 20 practice trials and was required to attain an accuracy above 60% to proceed to the formal experiment.

**Figure 1:**
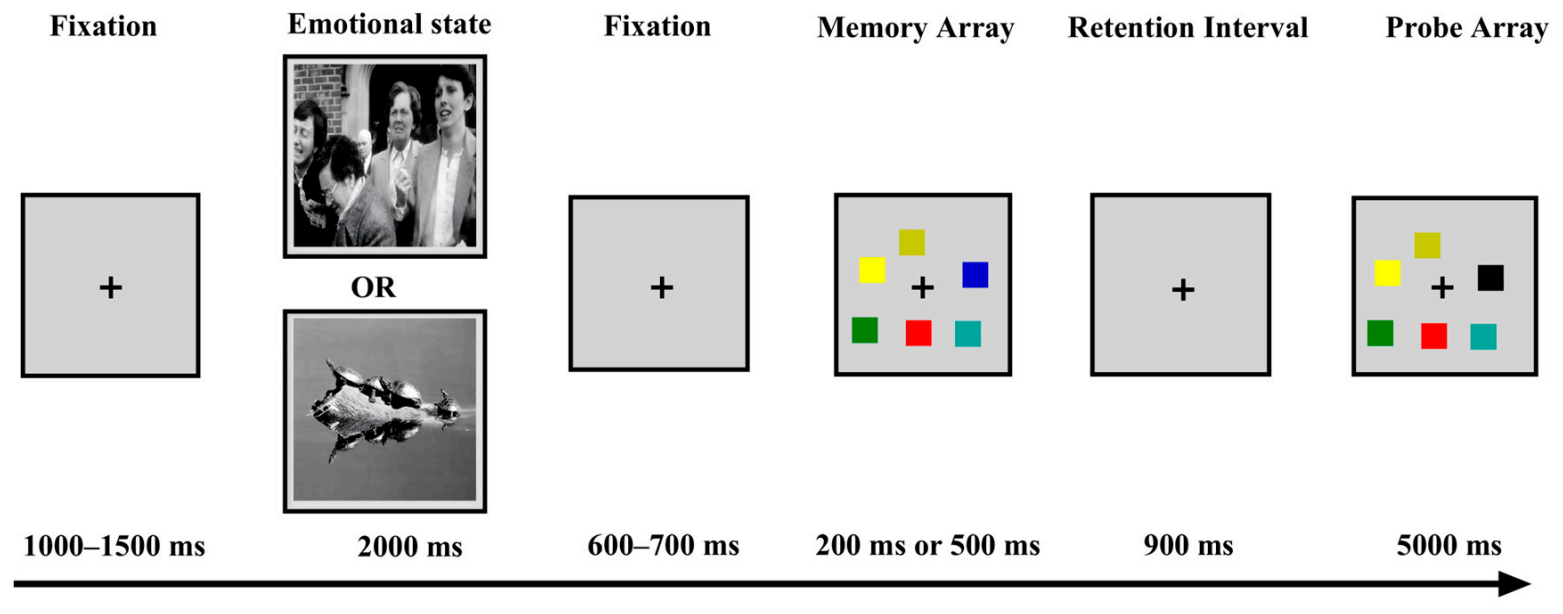
Flowchart of Experiment 1. Note. In the color-block condition, six color blocks were presented; in the probe array, the colors differed from those in the memory array.

As shown in Figure 1, participants were required to complete an emotion-eliciting change detection task. In the task, a fixation point was first presented at the center of the screen for 1000–1500 ms, followed by an emotion-eliciting picture (negative or neutral) for 2000 ms. After the picture disappeared, a fixation point was presented for a randomly varying duration of 600–700 ms, followed by a memory array of 2, 4, or 6 items for 200 ms or 500 ms. After the memory array disappeared, a “+” fixation point was presented at the center of the screen for 900 ms, and finally a probe array was presented. In the probe array, participants judged whether the currently presented color was the same as the previously presented color, pressing “F” for same and “J” for different (the experiment was set so that at most one item changed color), and they were required to respond within 5000 ms. Each probe item appeared at the location corresponding to its memory-array item. Participants responded only to whether a color had changed; spatial location was not a response-relevant dimension, although the memory–probe location correspondence could serve as an incidental retrieval cue.

#### 2.2.4. Data Analysis and Results

Data analysis was performed using IBM SPSS Statistics 26. Wilcoxon signed-rank tests were conducted separately on the valence and arousal ratings of the negative and neutral pictures in Experiment 1. A significant difference in valence was observed between the negative pictures (M = 2.52, SE = 0.82) and the neutral pictures (M = 5.50, SE = 0.75), Z = −6.15, *p* < 0.001; the difference in arousal between the negative pictures (M = 6.44, SE = 1.21) and the neutral pictures (M = 4.11, SE = 1.44) was also significant, Z = −5.76, *p* < 0.001. For the early and late stages of resource allocation, respectively, repeated-measures analyses of variance (ANOVAs) with a 2 (emotional state: negative, neutral) × 3 (cognitive load: low (2 items), high (4 items), excessive (6 items)) design were conducted separately on the K values, accuracy, and response times of the change detection task. When a significant interaction was observed, comparisons between the negative and neutral conditions at individual cognitive-load levels were conducted as follow-up simple-effects analyses to describe the interaction. Bonferroni adjustment was applied to the *p*-values of these multiple follow-up comparisons. The separate analyses for the 200 ms and 500 ms conditions were theoretically motivated by the two-phase model of VWM resource allocation. Specifically, the 200 ms condition was used to operationalize the early, predominantly stimulus-driven stage of resource allocation, whereas the 500 ms condition was used to operationalize the later stage, during which resource allocation may be more flexibly adjusted according to task demands. Accordingly, the present analyses were designed to examine the emotion–cognitive-load relation within each theoretically defined resource-allocation stage. Both emotional state and cognitive load were within-subjects variables. Prior to the response time analysis, error trials (11.39%) and trials exceeding 3 standard deviations (1.34%) were excluded (Pang et al., 2023).

##### Formula K

**Figure 2:**
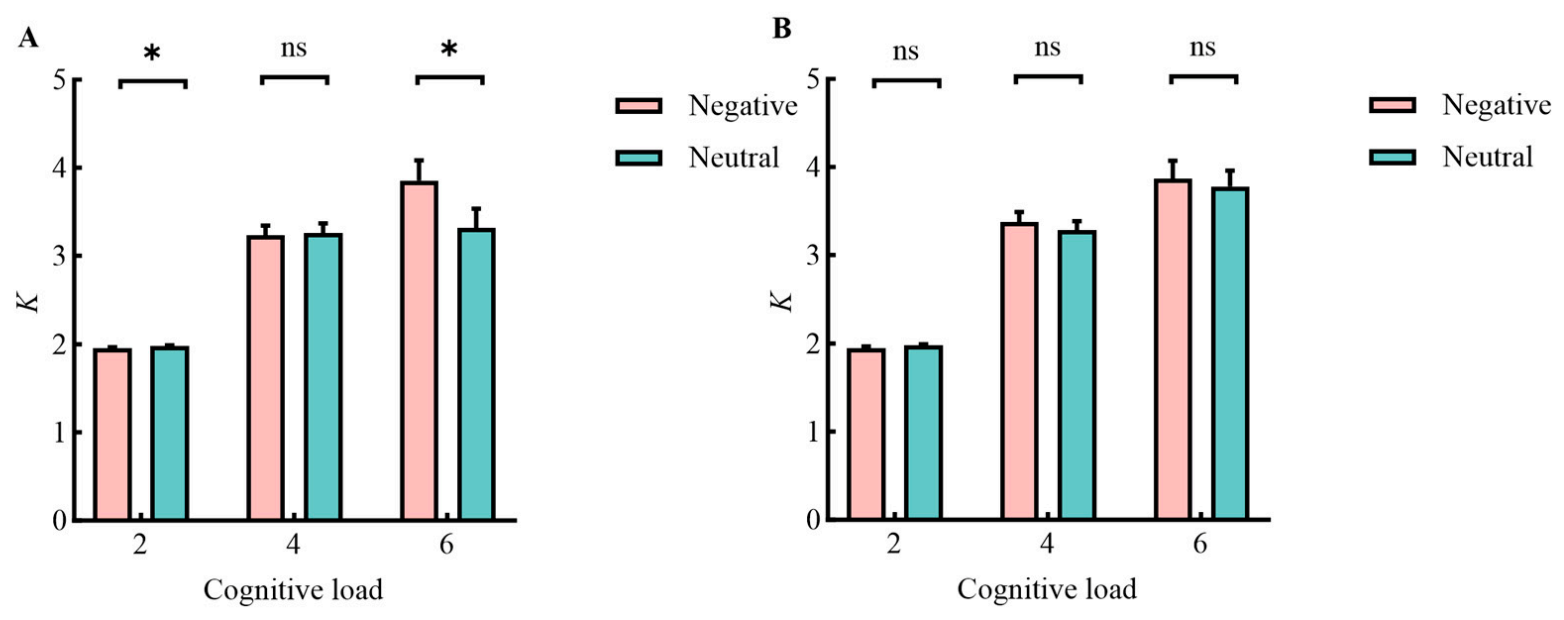
K-value results for emotional state under different cognitive-load conditions in Experiment 1. (**A**) The results at the early stage of resource allocation, and (**B**) the results at the late stage of resource allocation. Note. The error bars in the bar charts represent the standard error of the mean for each condition. * *p* < 0.05; ns indicates no statistically significant difference (*p* ≥ 0.05).

At 200 ms, the analysis of K-values revealed a significant main effect of emotional state, F (1, 34) = 4.80, *p* = 0.035, η*_p_*^2^ = 0.12, 95% CI [−0.31, −0.01], with K-values significantly higher in the negative emotional condition (M = 3.01, SE = 0.10) than in the neutral condition (M = 2.85, SE = 0.10). The main effect of cognitive load was also significant, F (1.25, 42.51) = 66.05, *p* < 0.001, η*_p_*^2^ = 0.66. Moreover, the interaction between emotional state and cognitive load reached significance, F (1.25, 42.36) = 5.04, *p* = 0.023, η*_p_*^2^ = 0.13 (see Figure 2). Simple-effects analyses showed that under low cognitive load, K-values were significantly lower in the negative emotional state (M = 1.94, SE = 0.02) than in the neutral state (M = 1.98, SE = 0.01), F (1, 34) = 5.99, *p* = 0.020, η*_p_*^2^ = 0.15, 95% CI [0.01, 0.07]. Under high cognitive load, no significant difference in K-values emerged between the negative (M = 3.24, SE = 0.11) and neutral (M = 3.26, SE = 0.11) emotional states, F (1, 34) = 0.05, *p* = 0.833, η*_p_*^2^ = 0.01, 95% CI [−0.19, 0.24]. Under excessive cognitive load, however, K-values were significantly higher in the negative emotional state (M = 3.86, SE = 0.23) than in the neutral state (M = 3.32, SE = 0.22), F (1, 34) = 6.15, *p* = 0.018, η*_p_*^2^ = 0.15, 95% CI [−0.98, −0.10].

At 500 ms, the K-value analysis revealed no significant main effect of emotional state, F (1, 34) = 0.42, *p* = 0.523, η*_p_*^2^ = 0.01, 95% CI [−0.20, 0.10]. In contrast, the main effect of cognitive load was significant, F (1.60, 54.36) = 95.96, *p* < 0.001, η*_p_*^2^ = 0.74. The interaction between emotional state and cognitive load was not significant, F (1.27, 43.26) = 0.28, *p* = 0.655, η*_p_*^2^ = 0.01 (as shown in Figure 2).

##### Accuracy Results

**Figure 3:**
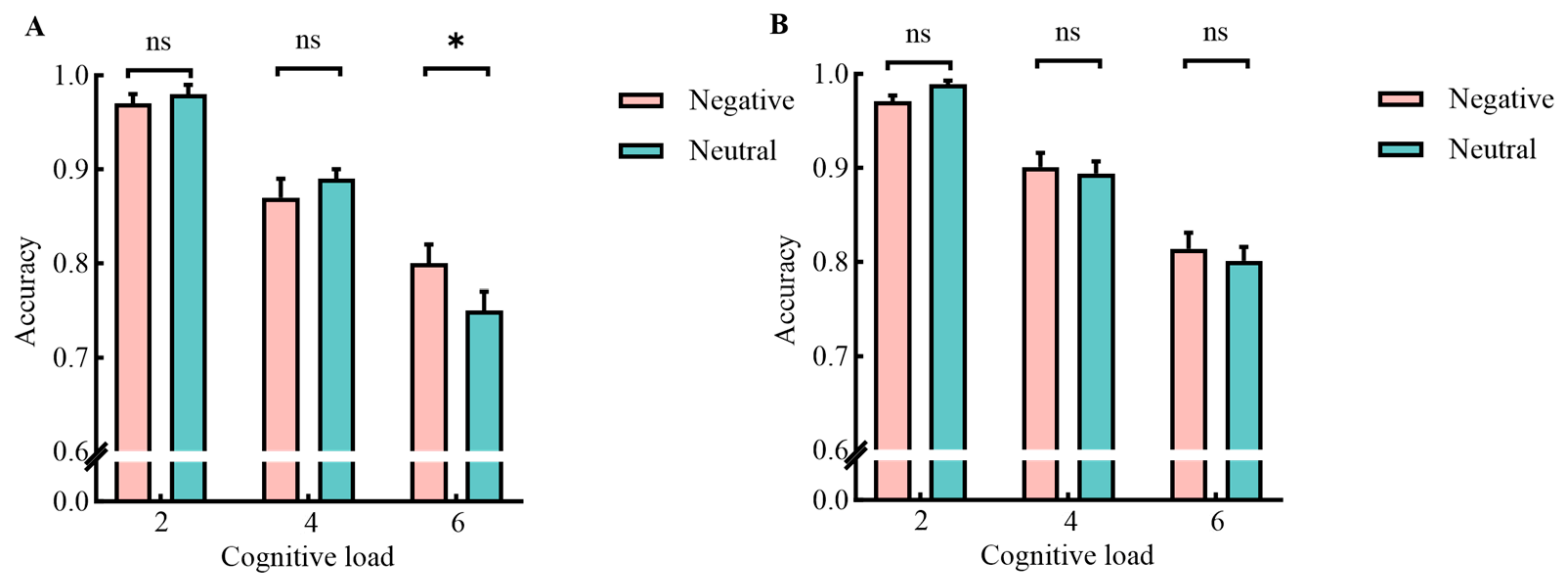
Accuracy results for emotional state under different cognitive-load conditions in Experiment 1. (**A**) The results at the early stage of resource allocation, and (**B**) the results at the late stage of resource allocation. Note. The error bars in the bar charts represent the standard error of the mean for each condition. * *p* < 0.05; ns indicates no statistically significant difference (*p* ≥ 0.05).

At 200 ms, the accuracy results showed that the main effect of emotional state was not significant, F (1, 34) = 0.71, *p* = 0.404, η*_p_*^2^ = 0.02, 95% CI [−0.02, 0.01], whereas the main effect of cognitive load was significant, F (1.66, 56.51) = 119.04, *p* < 0.001, η*_p_*^2^ = 0.78. The interaction between emotional state and cognitive load was also significant, F (1.65, 56.06) = 4.92, *p* = 0.015, η*_p_*^2^ = 0.13. Simple-effects analyses indicated (as illustrated in Figure 3) that under low cognitive load, there was no significant difference in accuracy between the negative emotional state (M = 0.97, SE = 0.01) and the neutral emotional state (M = 0.98, SE = 0.01), F (1, 34) = 0.86, *p* = 0.361, η*_p_*^2^ = 0.03, 95% CI [−0.01, 0.02]. Similarly, under high cognitive load, no significant difference emerged between the negative (M = 0.87, SE = 0.02) and neutral (M = 0.89, SE = 0.01) emotional states, F (1, 34) = 1.02, *p* = 0.319, η*_p_*^2^ = 0.03, 95%CI [−0.01, 0.04]. However, under excessive cognitive load, accuracy was significantly higher in the negative emotional state (M = 0.80, SE = 0.02) than in the neutral emotional state (M = 0.75, SE = 0.02), F (1, 34) = 5.26, *p* = 0.028, η*_p_*^2^ = 0.13, 95% CI [−0.08, −0.01].

At 500 ms, the accuracy results showed no significant main effect of emotional state, F (1, 34) = 0.02, *p* = 0.890, η*_p_*^2^ = 0.01, 95% CI [−0.02, 0.01], while the main effect of cognitive load was significant, F (2, 68) = 79.97, *p* < 0.001, η*_p_*^2^ = 0.70. The interaction effect between emotional state and cognitive load was not significant (as shown in Figure 3), F (2, 68) = 1.51, *p* = 0.229, η*_p_*^2^ = 0.04.

##### Reaction Time

At 200 ms, the response time results revealed no significant main effect of emotional state (negative emotional state: M = 697 ms, SE = 27 ms; neutral emotional state: M = 697 ms, SE = 25 ms), F (1, 34) = 0.01, *p* = 0.963, η*_p_*^2^ = 0.01, 95% CI [−13.24, 13.87]. In contrast, the main effect of cognitive load was significant (low cognitive load: M = 633 ms, SE = 24 ms; high cognitive load: M = 718 ms, SE = 27 ms; excessive cognitive load: M = 741 ms, SE = 27 ms), F (2, 68) = 54.55, *p* < 0.001, η*_p_*^2^ = 0.62. The interaction effect between emotional state and cognitive load was not significant, F (2, 68) = 0.96, *p* = 0.388, η*_p_*^2^ = 0.03.

At 500 ms, the response time results again showed no significant main effect of emotional state (negative emotional state: M = 694 ms, SE = 23 ms; neutral emotional state: M = 700 ms, SE = 25 ms), F (1, 34) = 1.00, *p* = 0.325, η*_p_*^2^ = 0.03, 95% CI [−6.73, 19.72], while the main effect of cognitive load was significant (low cognitive load: M = 637 ms, SE = 23 ms; high cognitive load: M = 706 ms, SE = 25 ms; excessive cognitive load: M = 747 ms, SE = 26 ms), F (1.39, 47.27) = 51.24, *p* < 0.001, η*_p_*^2^ = 0.60. The interaction between emotional state and cognitive load was not significant, F (1.57, 53.28) = 0.12, *p* = 0.840, η*_p_*^2^ = 0.01.

#### 2.2.5. Discussion

Experiment 1 examined the association between negative emotion, cognitive load, and VWM performance under the 200 ms and 500 ms memory-array presentation conditions. The results showed that the behavioral pattern associated with negative versus neutral emotion differed across cognitive-load conditions and presentation-duration conditions.

At the early stage of resource allocation (200 ms) under low cognitive load, negative emotion significantly reduced the estimated VWM capacity, as reflected by a lower K value in the negative emotional condition than in the neutral condition (1.94 vs. 1.98). Although the corresponding difference in accuracy did not reach statistical significance, this does not necessarily contradict the K-based finding, as K provides a specific estimate of the amount of information maintained in VWM. Taken together, these results suggest that during the early stage of processing, negative emotion may interfere with the allocation of limited cognitive resources under low cognitive load, resulting in a reduction in estimated VWM capacity. This result is consistent with previous findings (Figueira et al., 2017). However, under excessive cognitive load, the VWM performance of individuals in a negative emotional state was significantly enhanced—a result that has been rarely reported in prior research and is even inconsistent with the conclusions of some similar studies. We speculate that there are two reasons for this. First, most existing studies on the effect of negative emotion on VWM performance confined cognitive load within the range of VWM capacity without exceeding it; second, existing relevant studies have not thoroughly examined the influence of the resource-allocation stage under excessive cognitive load. In particular, previous findings indicate that basic features of memory items, such as color, shape, and location, are typically processed automatically at the early stage of VWM processing, and among these, location information is especially important for the process by which VWM binds features to objects (H. Chen & Wyble, 2015; Pertzov & Husain, 2013). However, the influence of location information gradually weakens over time (Treisman & Zhang, 2006), and negative emotion can enhance the processing of location information (Pinheiro et al., 2019).

We speculate that the emergence of the Experiment 1 results is closely related to the effects of the resource-allocation stage and location information. Under excessive cognitive load, the VWM performance under negative emotion was significantly better than under the neutral emotional state only at the early stage of resource allocation. This result also indicates that individuals in a negative emotional state, when confronted with excessive cognitive load, are more inclined to utilize location information to accomplish the subsequent memory task; the reason this result disappeared at the late stage of resource allocation may be that when the memory stimuli are presented for a sufficiently long duration, individuals tend to withdraw more attentional resources from the initial location information and invest them in processing item features (e.g., color, shape), thereby achieving better performance in possible subsequent fine-grained tasks.

Furthermore, in Experiment 1, participants were not required to memorize the locations of the items, yet under the early stage of resource allocation and excessive cognitive load, negative emotion enhanced VWM performance. The key to this result may be that during the brief memory processing, individuals automatically prioritize location information and apply it at the retrieval stage of memory representations. To verify this speculation, we conducted Experiment 2.

## 3. Experiment 2

### 3.1. Purpose

To verify the critical role of location information in the Experiment 1 results obtained at the early stage of resource allocation, Experiment 2 adopted essentially the same research paradigm as Experiment 1, but manipulated the presentation positions of the probe array so that they differed from those of the memory array. For example, under low cognitive load (two memory items), the positions at which the memory stimuli appeared in the probe array were all positions that had not appeared in the memory array. If the hypothesis underlying the Experiment 1 results holds, then under excessive cognitive load in Experiment 2, individuals in a negative emotional state at the early stage of resource allocation would be unable to utilize the automatically processed location information to enhance VWM performance.

### 3.2. Method

Forty adult students from Guizhou Normal University were randomly recruited. An a priori sample-size calculation was conducted using G*Power 3.1 for a repeated-measures ANOVA. The calculation assumed a medium effect size of Cohen’s f = 0.25, an alpha level of 0.05, and a target power of 0.85, yielding a minimum required sample size of 21 participants. The sample included 21 females, with a mean age of 20.47 ± 0.29 years. Data from four participants whose accuracy was below 60% were excluded, leaving data from 36 adult participants for the final analysis. All participants had normal or corrected-to-normal vision, no color blindness or color weakness, and were right-handed. They received monetary compensation upon completion of the experiment. This study was approved by the Committee for the Protection of Human Subjects at the School of Psychology, Guizhou Normal University (approval number: GZNUPSY.N.202312E [0021]).

The task procedure of Experiment 2 was otherwise identical to that of Experiment 1. Participants continued to judge whether a color had changed, but the probe items were presented at locations that had not been occupied by items in the preceding memory array. Thus, Experiment 2 removed memory–probe location congruence without changing the response-relevant feature from color to location.

### 3.3. Data Analysis and Results

The data analysis procedure for Experiment 2 was the same as for Experiment 1. Prior to the response time analysis, error trials (13.82%) and trials exceeding three standard deviations (1.00%) were excluded.

#### 3.3.1. Formula K

**Figure 4:**
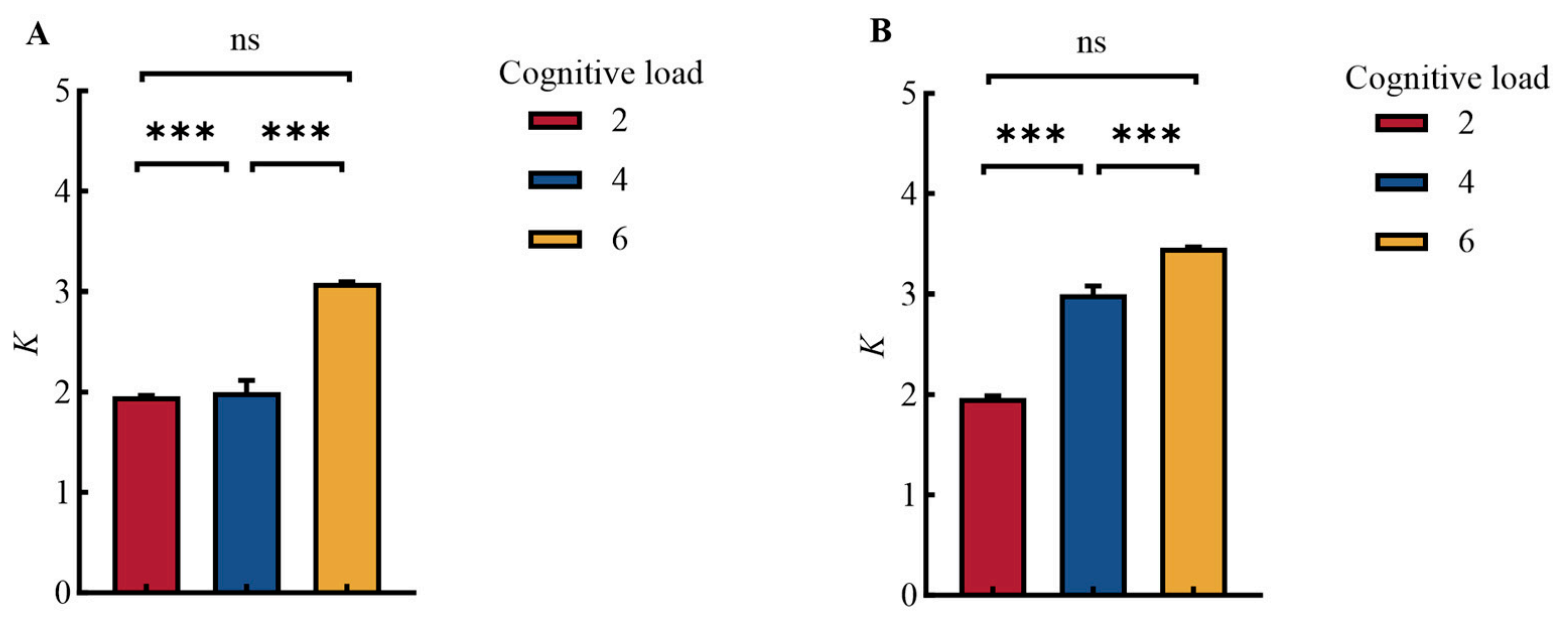
K-value results for cognitive load in Experiment 2. (**A**) The K-value results under the 200 ms condition, and (**B**) the K-value results under the 500 ms condition. The bars represent estimated marginal means for each cognitive-load condition averaged across the negative and neutral emotional states. Note. The error bars in the bar charts represent the standard error of the mean for each condition. *** *p* < 0.001; ns indicates no statistically significant difference (*p* ≥ 0.05).

At 200 ms, the K-value analysis revealed no significant main effect of emotional state, F (1, 35) = 1.17, *p* = 0.287, η*_p_*^2^ = 0.03, 95% CI [−0.07, 0.24], a significant main effect of cognitive load (see Figure 4), F (2, 70) = 47.63, *p* < 0.001, η*_p_*^2^ = 0.58, and a non-significant emotional state × cognitive load interaction, F (1.27, 44.33) = 2.27, *p* = 0.134, η*_p_*^2^ = 0.06.

At 500 ms, the K-value results similarly showed no significant main effect of emotional state, F (1, 35) = 0.32, *p* = 0.576, η*_p_*^2^ = 0.01, 95% CI [−0.14, 0.24]. However, the main effect of cognitive load was significant (as shown in Figure 4), F (1.41, 49.22) = 77.46, *p* < 0.001, η*_p_*^2^ = 0.69. The interaction effect between emotional state and cognitive load was not significant, F (1.36, 47.44) = 0.94, *p* = 0.364, η*_p_*^2^ = 0.03.

#### 3.3.2. Accuracy Results

**Figure 5:**
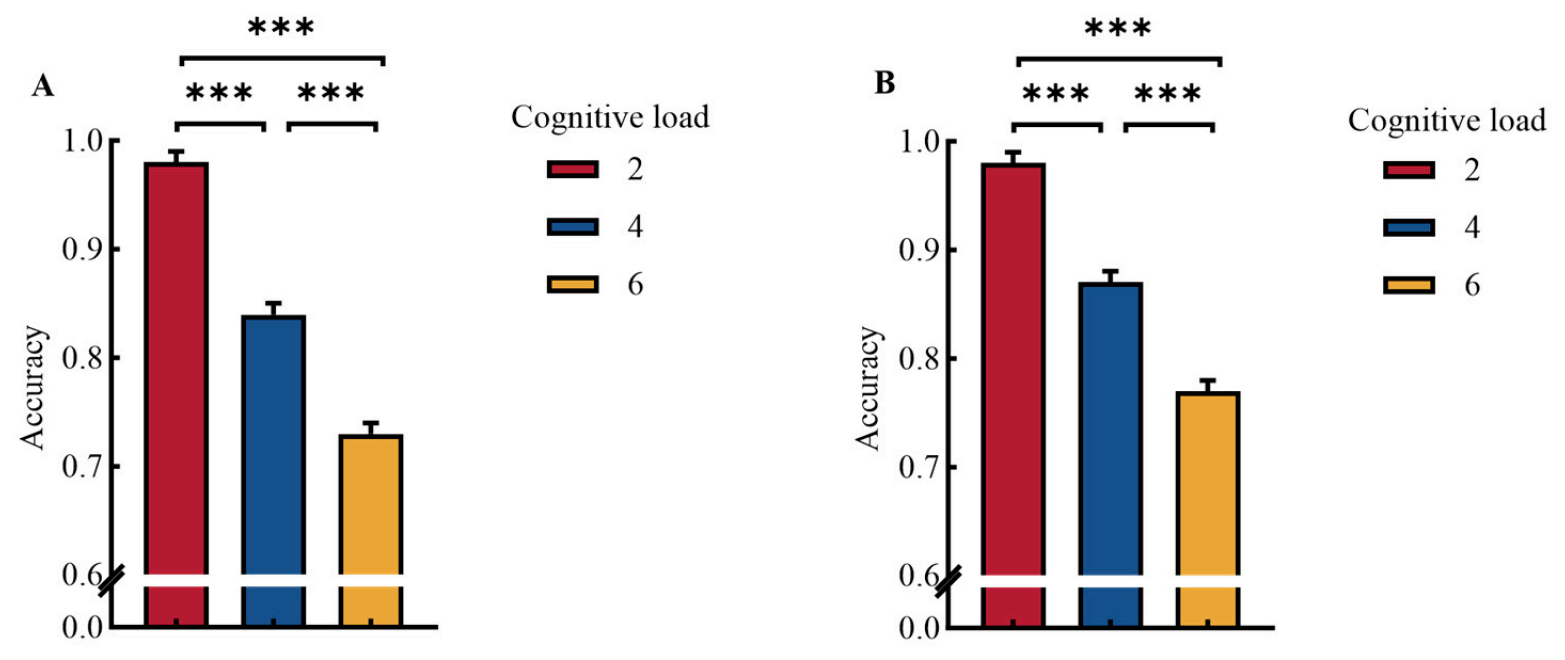
Accuracy results for cognitive load in Experiment 2. (**A**) The accuracy results under the 200 ms condition, and (**B**) the accuracy results under the 500 ms condition. The bars represent estimated marginal means for each cognitive-load condition averaged across the negative and neutral emotional states. Note. The error bars in the bar charts represent the standard error of the mean for each condition. *** *p* < 0.001; ns indicates no statistically significant difference (*p* ≥ 0.05).

At 200 ms, the accuracy results showed that the main effect of emotional state was not significant, F (1, 35) = 0.57, *p* = 0.457, η*_p_*^2^ = 0.02, 95% CI [−0.01, 0.02]; the main effect of cognitive load was significant (as shown in Figure 5), F (2, 70) = 193.81, *p* < 0.001, η*_p_*^2^ = 0.85. The interaction effect between emotional state and cognitive load was not significant, F (1.42, 49.79) = 1.05, *p* = 0.337, η*_p_*^2^ = 0.03.

At 500 ms, the accuracy results showed that the main effect of emotional state was not significant, F (1, 35) = 0.23, *p* = 0.636, η*_p_*^2^ = 0.01, 95% CI [−0.02, 0.02]; the main effect of cognitive load was significant (as illustrated in Figure 5), F (2, 70) = 143.60, *p* < 0.001, η*_p_*^2^ = 0.80. The interaction effect between emotional state and cognitive load was not significant, F (1.62, 56.81) = 1.62, *p* = 0.211, η*_p_*^2^ = 0.04.

#### 3.3.3. Reaction Time

At 200 ms, the response time analysis revealed no significant main effect of emotional state (negative emotional state: M = 930 ms, SE = 35 ms; neutral emotional state: M = 933 ms, SE = 36 ms), F (1, 35) = 0.05, *p* = 0.829, η*_p_*^2^ = 0.01, 95% CI [−22.02, 27.32]. In contrast, the main effect of cognitive load was significant (low cognitive load: M = 703 ms, SE = 21 ms; high cognitive load: M = 1000 ms, SE = 42 ms; excessive cognitive load: M = 1089 ms, SE = 50 ms), F (1.41, 49.53) = 72.33, *p* < 0.001, η*_p_*^2^ = 0.67. The interaction between emotional state and cognitive load did not reach significance, F (2, 70) = 0.66, *p* = 0.520, η*_p_*^2^ = 0.02.

At 500 ms, the response time data again showed a nonsignificant main effect of emotional state (negative emotional state: M = 940 ms, SE = 35 ms; neutral emotional state: M = 933 ms, SE = 36 ms), F (1, 35) = 0.45, *p* = 0.506, η*_p_*^2^ = 0.01, 95% CI [−29.46, 14.81], while the main effect of cognitive load was significant (low cognitive load: M = 703 ms, SE = 20 ms; high cognitive load: M = 989 ms, SE = 42 ms; excessive cognitive load: M = 1117 ms, SE = 56 ms), F (1.18, 41.39) = 60.90, *p* < 0.001, η*_p_*^2^ = 0.64. The interaction between emotional state and cognitive load was also not significant, F (2, 70) = 1.13, *p* = 0.330, η*_p_*^2^ = 0.03.

### 3.4. Discussion

In Experiment 2, the presentation positions of the memory representations in the probe array were altered relative to those in the memory array. The results showed that under excessive cognitive load, the facilitative effect of negative emotion on VWM performance at the early stage of resource allocation disappeared. The results indicated that the relation between negative emotion and VWM performance varied across cognitive-load conditions and showed different patterns across the early and late resource-allocation stages and location-information conditions. In particular, under excessive cognitive load, higher VWM performance in the negative than neutral condition was observed during the early resource-allocation stage when memory-array and probe-array locations were congruent. This pattern suggests that the resource-allocation stage and location congruence may be important conditions for understanding the effect of negative emotion on VWM performance. In Experiment 2, although the presentation positions of the probe array were altered to differ from those of the memory array, participants may still have automatically processed spatial information during the presentation of the memory array. However, the location information encoded at that stage no longer facilitated performance on the probe-array task.

## 4. General Discussion

Through two experiments, the present study investigated the joint effects of negative emotion and cognitive load on VWM across theoretically defined resource-allocation stages and location-information conditions. The results showed that the association between negative emotion and VWM performance varied across cognitive-load conditions and differed in pattern between the early and late resource-allocation stages operationalized by 200 ms and 500 ms memory-array presentations. In particular, under excessive cognitive load, higher VWM performance in the negative than neutral condition was observed in the 200 ms condition when memory-array and probe-array locations were congruent. This pattern is consistent with the possibility that the processing of location information may be relevant under these conditions; however, the present study did not directly measure the underlying processing mechanism.

Previous studies have reported mixed effects of negative emotion on VWM, with findings suggesting that negative emotion can either impair or facilitate VWM performance depending on cognitive load and processing conditions. However, the mechanisms underlying these inconsistent effects remain unclear. The present study extends previous research by demonstrating that the influence of negative emotion on VWM is context-dependent rather than uniformly beneficial or detrimental. Specifically, negative emotion impaired VWM under low cognitive load but enhanced VWM performance under excessive cognitive load when the early stage of resource allocation and location information were preserved. These findings highlight that cognitive load, the stage of resource allocation, and location information availability jointly determine how emotional states influence VWM performance, providing a more comprehensive account of emotion–cognition interactions.

### 4.1. The Effect of Negative Emotional State on VWM Processing

The results of the present study indicate that the influence of negative emotion on VWM performance is neither unidirectional nor stable, but rather exhibits clear context-dependence. In Experiment 1, the K value and accuracy during the early phase of resource allocation were significantly higher under negative emotion than under neutral emotion, indicating that negative emotion may facilitate VWM. This finding aligns with previous research (Zhang et al., 2020), in which negative, neutral, and positive emotions were induced through video clips and a change detection paradigm was employed alongside electroencephalography. That study demonstrated that negative emotion significantly enhanced VWM performance, a pattern consistently replicated in Experiment 1 of the present study. However, in Experiment 2, after the probe-array positions were altered, this effect disappeared, and the overall main effect of emotional state was also non-significant, indicating that the facilitative effect of negative emotion on VWM performance is not universally present, but rather depends on specific processing conditions, in particular the early stage of resource allocation and the stability of visuospatial position.

### 4.2. The Joint Effect of Negative Emotional State and Cognitive Load on VWM

Under the low cognitive-load condition in Experiment 1, negative emotion at the early stage of resource allocation was associated with a significantly lower K estimate, suggesting a reduction in estimated VWM capacity. Although the corresponding difference in accuracy did not reach statistical significance, the overall pattern suggests that negative emotion may interfere with early VWM processing under low cognitive load. This is consistent with the Resource Allocation Theory: under low-load memory conditions, individuals have abundant cognitive resources, and negative emotion readily causes attentional distraction, occupying part of the resources that should be devoted to processing the memory task (Kahneman, 1973). This phenomenon accords with the assumption of the PLT (Z. Chen & Wang, 2025; Lavie, 2005), namely that when individuals perceive a low task load, they automatically increase their attention to and processing of emotion-related information, compromising their concentration on the memory task; another explanation is that after experiencing negative emotion, individuals consume cognitive resources on rumination, resources that would otherwise be used to execute the target task (Curci et al., 2013).

This result also converges with neurophysiological evidence. Chouinard-Gaouette and Blanchette (2025) revealed a neural mechanism that is consistent with the behavioral findings of the present study. Under high cognitive load, negative emotion had no significant effect on VWM, a result that likewise supports the PLT: that is, when task load increases, attentional resources are almost entirely occupied by the target task, thereby reducing the processing of negative emotional information and weakening its interference. The neuroimaging study by Chouinard-Gaouette and Blanchette (2025) also showed that under high cognitive load, negative emotion had no significant effect on P300 amplitude, consistent with the present behavioral results. However, the present study found that under excessive cognitive load, the influence of negative emotion on VWM exhibited a different pattern.

Under the excessive cognitive-load condition in Experiment 1, negative emotion did not impair VWM performance at the early stage of resource allocation; on the contrary, it facilitated VWM performance. This result may stem from an alertness-enhancement mechanism induced by negative emotion. According to the Yerkes–Dodson law (Sinha, 2024; Yerkes & Dodson, 1908), in highly challenging task contexts, moderate emotional arousal helps optimize the individual’s cognitive state. When task demands exceed the conventional limit of VWM capacity, negative emotion may prompt the individual to enter a state of stress preparedness, thereby enhancing the degree of attentional focus and preferentially initiating the automatic processing of key task cues (such as location information). This mechanism enables the individual, even under conditions of extreme cognitive resource scarcity, to achieve a temporary improvement in processing efficiency by optimizing resource allocation.

### 4.3. The Processing of Location Information in VWM Tasks

The present study also found that the joint effect of negative emotion and cognitive load on VWM is influenced by the processing of location information of memory items. Although participants were not required to actively memorize location information, in Experiment 1, when the positions of memory items and probe items were congruent, individuals may have spontaneously utilized spatial cues to optimize the processes of memory encoding and retrieval. This finding is consistent with the feature binding theory, namely that visual features and location information are automatically integrated into a unified representation in working memory (Cao et al., 2026). Behavioral studies have also shown that spatial cues can effectively improve memory efficiency through location encoding strategies, and that negative emotion enhances the processing sensitivity to location information (Pinheiro et al., 2019).

Under low cognitive load in Experiment 1, the memory and probe arrays preserved the same item locations. Although location was not the response-relevant feature, this spatial correspondence could provide an incidental cue during color retrieval. Within this condition, negative emotion may have competed for resources available to the memory task, which may account for the small decrease in the K estimate. This finding is consistent with the recent study by Chouinard-Gaouette and Blanchette, namely that under low working-memory load, emotional stimuli receive more thorough processing.

In Experiment 2, the probe items were relocated while color remained the response-relevant feature. This manipulation removed the spatial correspondence between the memory and probe items rather than requiring participants to detect a location change. Under low cognitive load, the small emotion-related difference in K observed in Experiment 1 was no longer detected. Under high cognitive load, regardless of whether positions were congruent, no significant effect of negative emotion was observed. This finding aligns with the predictions of CLT, which posits that when a task demands substantial cognitive resources, the system prioritizes the execution of the core task while suppressing task-irrelevant emotional processing. This moderating effect has received further support in recent research, which demonstrates that high cognitive load can effectively reduce attentional bias toward negative stimuli (Luo, 2025). Under excessive cognitive load, when memory item positions were congruent, individuals’ cognitive resources were already oversaturated and occupied. In this situation, negative emotion can evoke a stress response, bringing the individual into a highly alert “optimal arousal” state (Soares et al., 2026), concentrating more attention on the current memory task and utilizing location information to improve task performance, thereby enhancing VWM performance; whereas when memory item positions were incongruent, individuals not only had to remember the item contents but also had to process the information about positional changes, and the complexity of the task increased markedly. Although negative emotion can theoretically heighten alertness, its alerting effect may be offset by the increased difficulty of the task itself, and may even aggravate the individual’s sense of task frustration, thereby impairing VWM performance.

### 4.4. The Effect of the Resource-Allocation Stage

The present study examined whether the pattern of association between negative emotion, cognitive load, and VWM performance differed across the theoretically defined early and late resource-allocation stages, operationalized by 200 ms and 500 ms memory-array presentations.

In Experiment 1, individuals under excessive cognitive load and in a negative emotional state performed better on the VWM task, and the facilitative effect of location information on the excessive cognitive load memory task emerged only at the early stage of resource allocation. The present study contends that the necessary condition for this facilitative effect is the automatically processed location information. This is consistent with previous research: Treisman and Zhang (2006), by varying stimulus features such as color, shape, and location and using different probe types, tested participants’ memory for features and bindings, and found that location information is crucial for binding features and objects in VWM, especially at the early stage of memory formation, a process that occurs automatically and without voluntary control. Moreover, the influence of location information gradually diminishes over time. Pertzov and Husain (2013) further used a continuous response scale to examine the types of reporting errors when objects were presented at the same or different spatial locations, and likewise found that location information is automatically maintained during memory, even when task-irrelevant. In a recent study, Goldenhaus-Manning et al. (2023) also found that, compared with other intrinsic features (such as color and shape), location information guides attention in a manner that facilitates memory for the target.

In Experiment 1, participants were not required to memorize the positions of memory items, yet the memory items appeared at congruent positions in both the memory array and the probe array; during the process of memorizing colors, individuals automatically bound the colors to the item positions and used them as reference information at the probe interface to complete the change detection task. However, location information assisting cognitive processing must satisfy certain conditions (Hollingworth & Henderson, 2002): in their study, they found that the influence of location information in VWM depends on the allocation of attention, and Pinheiro et al. (2019), in studying how spatial position and emotion affect sound perception, found that compared with neutral emotion, negative emotion enhanced the recognition of location information. Therefore, after Experiment 2 eliminated the information about position congruence between the preceding and following arrays, the facilitating effect of location information disappeared.

### 4.5. Limitations

The present study has several limitations that should be acknowledged. First, task load was manipulated in a controlled laboratory setting, which, although effective in minimizing extraneous variables, may not fully capture the complexity of cognitive demands in real-world contexts. Factors such as environmental noise, task variability, and multiple sources of interference were not accounted for, potentially limiting the ecological validity of the findings. Second, because negative emotional stimuli typically involve both negative valence and heightened arousal, the current design cannot fully disentangle the independent contributions of these two emotional components. Future research could address this by manipulating valence and arousal separately to clarify their respective effects. Third, the level of excessive cognitive load examined in this study was relatively constrained and did not encompass the extreme overload conditions individuals may encounter in daily life. Subsequent studies could investigate how varying intensities of excessive load interact with negative emotion to influence VWM. Finally, future work may benefit from incorporating multimodal measures—such as physiological indicators like skin conductance response and heart rate, along with neuroimaging data—to enhance the objectivity and precision of emotional assessment. In addition, the resource-allocation stage was operationalized through memory-array presentation duration rather than measured directly. Although this operationalization was theoretically based on the two-phase model of VWM resource allocation, presentation duration may also influence encoding completeness, attentional allocation, and strategy use. Future research should combine behavioral paradigms with process-sensitive measures, such as electrophysiological indices, to distinguish these possibilities more directly.

## 5. Conclusions

The present study investigated the impact of negative emotion on VWM and its relation to cognitive load across theoretically defined resource-allocation stages and location-information conditions. In Experiment 1, under the condition operationalizing the early resource-allocation stage (200 ms memory-array presentation) and with congruent memory-array and probe-array locations, negative emotion was associated with lower VWM performance under low cognitive load but higher VWM performance under excessive cognitive load. This pattern was not observed in the 500 ms condition or after probe-array locations were altered in Experiment 2. These findings indicate that the emotion–cognitive-load relation showed different patterns across the theoretically defined early and late resource-allocation stages and across location-information conditions.

## Data Availability

The original contributions presented in this study are included in the article. Further inquiries can be directed to the corresponding author.
